# Perception of AI-induced occupational replacement risk and withdrawal behavior: an identity threat perspective in the workplace

**DOI:** 10.3389/fpsyg.2026.1922666

**Published:** 2026-10-13

**Authors:** Hui Yang

**Affiliations:** Faculty of Education, East China Normal University, Shanghai, China

**Keywords:** AI-induced occupational replacement risk perception, identity threat, job insecurity, professional identity, technological self-efficacy, withdrawal behavior, work meaningfulness

## Abstract

As artificial intelligence (AI) becomes increasingly integrated into the workplace, the risk that occupations will be replaced by AI has received widespread attention. Nevertheless, the underlying mechanisms linking AI-induced occupational replacement risk perception to individual withdrawal behaviors remain underexplored. Taking identity threat as an explanatory lens, this study integrates Identity Threat Theory (ITT) and Technology Threat Avoidance Theory (TTAT) to investigate the effect of occupational replacement risk perception on employee withdrawal behavior, with professional identity, work meaningfulness, and job insecurity serving as mediators and technological self-efficacy as a moderator. Two studies were conducted to test the theoretical model developed in this research. Study 1 developed and validated the AI-induced occupational replacement risk perception scale. Study 2 employed a three-wave time-lagged survey design to test the hypotheses. The results show that occupational replacement risk perception is positively associated with withdrawal behavior through decreased professional identity and work meaningfulness as well as increased job insecurity. Furthermore, these indirect effects are weaker among employees with higher technological self-efficacy. By illuminating the identity-threat mechanism that links AI-induced occupational replacement risk perception to employee behavior, this study opens the black box of how AI shapes employee responses, and it concludes with a discussion of the theoretical contributions and practical implications of these findings.

## Introduction

While the advancement of artificial intelligence (AI) technology has substantially boosted workplace productivity and organizational operational efficiency (Noy and Zhang, 2023), they have also had unpredictable impacts on occupations (Lent, 2018; Westman et al., 2021). In particular, the widespread application of generative AI has accelerated the disappearance of traditional occupations and further elevated the risk of occupational replacement (Bankins et al., 2024; Roos and Shroff, 2017). Previous research estimated that approximately 47% of existing occupations would face high risks of automation-driven replacement within two decades (Frey and Osborne, 2017). Consistent with this prediction, recent evidence indicates that 32.8% of occupations were fully exposed to ChatGPT in 2023, with an additional 36.5% partially affected (Zarifhonarvar, 2024). Together, these findings demonstrate that AI technologies are profoundly reshaping occupational structures, changing the nature of work, and broadening the range of occupations vulnerable to technological replacement (Bankins et al., 2024; Haenlein and Kaplan, 2019).

Although previous studies in economics and sociology have developed objective indices of occupational replacement risk by various methods (Felten et al., 2018; Fossen and Sorgner, 2022; Frey and Osborne, 2017), few studies have examined individuals’ subjective perceptions of such risk. Even as workers increasingly perceive the existential threat that AI poses to their occupations, the downstream psychological and behavioral consequences of perceived replacement risk remain poorly understood, a gap this study addresses. More recently, research has further revealed the identity-related implications of AI, indicating that AI’s encroachment on core professional competencies can generate professional identity threat and prompt defensive responses (Zhou et al., 2025). Despite this accumulating evidence, existing research remains fragmented. Most studies examining isolated attitudinal or behavioral outcomes, while few have adopted a threat perspective to systematically theorize the mechanisms through which employees perceived occupational replacement risk translates into workplace withdrawal behavior.

To address this gap, the present study investigates how perceived AI-induced occupational replacement risk shapes employees’ behavioral responses by integrating Identity Threat Theory (ITT, Petriglieri, 2011) with Technology Threat Avoidance Theory (TTAT, Liang and Xue, 2009). ITT holds that individuals experience threat when they appraise an experience as indicating “potential harm to the value, meanings, or enactment of an identity” (Petriglieri, 2011), and each of these categories of identity threats represents a distinct potential structure with its own reflective indictors (George et al., 2023; MacKenzie et al., 2011). Because occupations constitute a fundamental source of individual identity (Thatcher and Zhu, 2006; Cardador and Pratt, 2006), the adoption of AI in the workplace may engender identity threat by weakening the uniqueness of human work and challenging employees’ identities and values. Mapping onto the three core attributes of identity, perceived occupational replacement risk may threaten different aspects of employees’ occupational identity: it may devalue a valued professional identity, erode the meaning associated with one’s work, and obstruct the continued enactment of that identity. Accordingly, professional identity threat, diminished work meaningfulness, and job insecurity can be understood as three proximal threat states corresponding to these categories. We therefore propose that perceived AI-induced occupational replacement risk threatens employees’ professional identity and work meaningfulness while heightening their sense of job insecurity.

Technology threat avoidance theory (TTAT) further specifies the psychological mechanism underlying the behavioral responses that follow. Originally developed to explain how individuals voluntarily avoid information technology threats, this theory posits that threat perceptions give rise to avoidance motivation and, ultimately, avoidance-oriented behaviors (Liang and Xue, 2009). This logic aligns closely with the two-stage structure of identity threat theory, which distinguishes threat appraisal from threat response (Petriglieri, 2011). When individuals face identity threats, they adopt protective responses aimed at reducing or eliminating the threat. In the workplace, behavioral withdrawal, such as voluntary turnover and absenteeism, represents a prototypical protective response through which employees’ distance themselves from a threatening environment (Hanisch and Hulin, 1991). In this sense, AI constitutes a potent sociotechnical threat that elicits protective cognitions and avoidance-oriented behavioral responses. We therefore argue that perceived AI-induced occupational replacement risk triggers withdrawal behavior when employees experience threats to their professional identity, work meaningfulness, and job security.

Whether replacement risk translates into withdrawal behavior largely depends on individuals perceived coping capacity, as coping-appraisal processes constitute important boundary conditions for threat-related responses. TTAT suggests that perceived threats do not automatically lead to avoidance motivation. Instead, this translation depends on coping appraisal, namely, whether individuals believe the threat can be effectively managed (Liang and Xue, 2009). Self-efficacy is central to this appraisal process. It refers to the belief in one’s capability to successfully perform challenging tasks (Bandura and Wessels, 1997). Identity threat theory holds a similar view, arguing that threat experience is contingent on appraised coping resources (Petriglieri, 2011). Building on these perspectives, we focus on technological self-efficacy, defined as employees’ belief in their ability to master new and technologically demanding tasks (McDonald and Siegall, 1992), which can help counteract the perceived threat posed by AI in the workplace (Alahmad and Robert, 2020; Craig et al., 2019). When facing AI as a perceived threat, strong technological self-efficacy supports both coping appraisal and self-protective behaviors (Chang et al., 2024). Those with higher technological self-efficacy are more likely to view AI-related challenges as opportunities for growth rather than threats (Yener et al., 2021). Therefore, we propose that it serves as a key boundary condition of the relationship between perceived occupational replacement risk and identity-threat experiences.

The current study makes contributions at the literature, theoretical, and practical levels. While the growing body of literature on artificial intelligence underscores the criticality of examining individual risk perceptions and advocates for exploring diverse forms of subjective risk (Huang and Rust, 2021), current studies largely overlooks the potential impact of technological advances on occupations (Bankins et al., 2024). In response to call for examining individuals’ responses to technological replacing human labor before technological advances destroy specific occupations (Granulo et al., 2019), this study investigates the underlying mechanism of perceived occupational replacement risk and withdrawal behavior. Theoretically, by integrating the ITT and TTAT as the identity threat theoretical framework, we explore how occupational replacement risk perception influence withdrawal behavior through undermining individual professional identity, erode their work meaningfulness, and amplifies feelings of job insecurity. This integrated model not only extends Petriglieri’s (2011) theoretical framework of threat-induced identity processes and George et al.’s (2023) contemporary typology of workplace identity threats but also advances the application of TTAT under AI context. Practically, with the rapid advances in technology, technological self-efficacy has also been explored in AI-related contexts (Kaltenegger, 2024), this study highlights technological self-efficacy as a strategic coping resource that equips employees to effectively navigate and mitigate the disruptions associated with technological change in the workplace. The full proposed model is presented in Figure 1.

**Figure 1:**
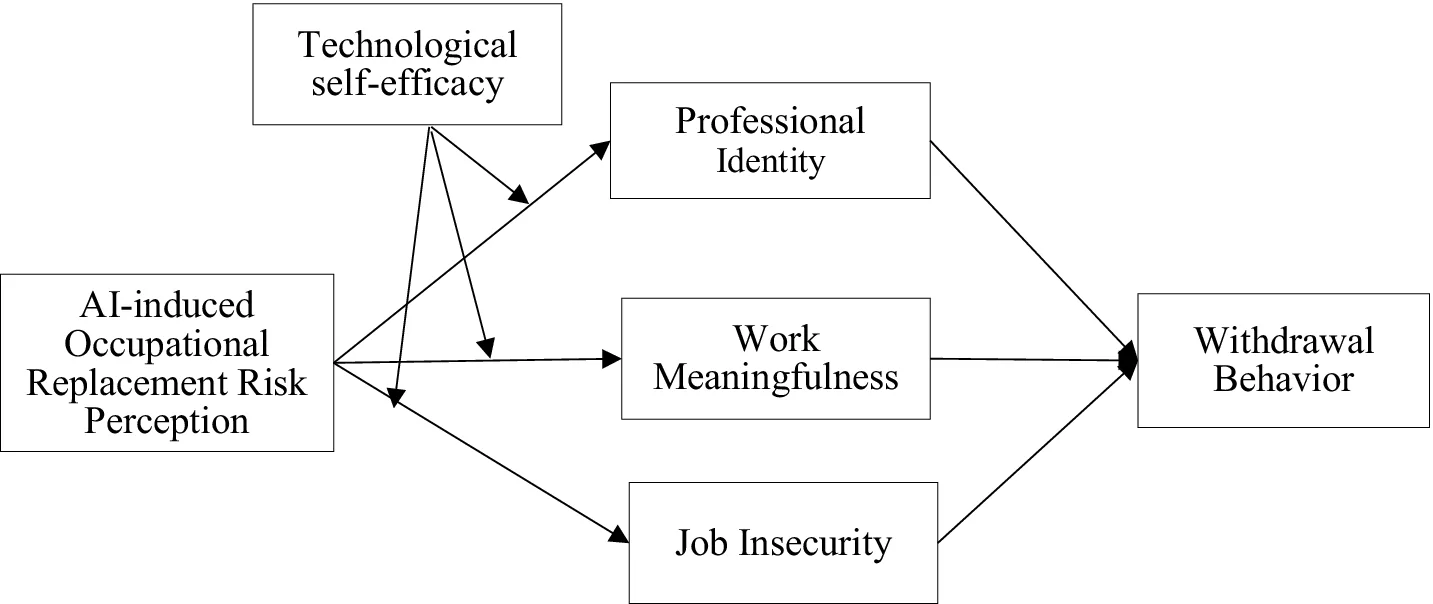
Hypothesized model.

## Theoretical foundations and hypotheses development

### AI-induced occupational replacement risk perception

Risk perception refers to an individual’s awareness of external risks, reflecting their subjective judgment and evaluation of existing or potential environmental threats (Rohrmann and Renn, 2000). Conceptually, risk perception comprises two fundamental dimensions: the perceived likelihood risk occurrence and the perceived severity of its potential consequences (Sjöberg, 2000; Wilson et al., 2019). In the workplace, risk perception typically arises in response to uncertainties such as technological disruption, organizational change, and role ambiguity (Neyazi et al., 2023). In this sense, we define AI-induced occupational replacement risk perception as an individual’s subjective appraisal of the likelihood that AI will replace his or her occupation and of the severity of the consequences of such replacement. This dual-component appraisal distinguishes construct validity of risk perception from related but distinct workplace AI constructs. Specifically, AI-induced occupational replacement risk perception is a threat appraisal that integrates evaluations of probability and consequence, whereas STARA awareness and AI awareness capture only the cognitive judgment that one’s job can be performed by technology (Brougham and Haar, 2020; Li et al., 2019), and AI anxiety captures a generalized affective response to AI that requires no appraisal of one’s own occupation (Wang and Wang, 2022). Accordingly, AI-induced occupational replacement risk perception constitutes a targeted, person-specific evaluative judgment that synthesizes likelihood and consequence, representing a structured threat assessment.

### Theoretical foundations

Identity threat theory (Petriglieri, 2011) holds that individuals experience threat when they appraise an experience as indicating “potential harm to the value, meanings, or enactment of an identity” (Petriglieri, 2011). An identity-threatening experience thus materializes in at least one of three registers: the esteem or worth attached to the identity (value), the understandings associated with it (meanings), or the activities and interactions that express it (enactment; Petriglieri, 2011; George et al., 2023). Importantly, George et al. (2023) clarify that identity threat is not an overarching latent construct with reflective subdimensions but a domain comprising three distinct types of experience, each constituting a separate construct. Accordingly, we conceptualize threats to professional identity, threats to meaningfulness, and job insecurity as three distinct manifestations of identity threat arising from perceived AI occupational replacement risk, and we model them as three parallel mechanisms through which this risk perception exerts its effects.

Technology threat avoidance theory (TTAT; Liang and Xue, 2009) explains why and how individuals avoid information technology threats, and it distinguishes two appraisal processes. In threat appraisal, users form threat perceptions from two beliefs, namely perceived susceptibility, the subjective probability that the technology will negatively affect them, and perceived severity, the perceived magnitude of the resulting consequences (Liang and Xue, 2009). In coping appraisal, users evaluate whether the threat is avoidable given their self-efficacy in applying safeguarding measures (Liang and Xue, 2009; Chen and Zahedi, 2016). Appraised threats subsequently generate avoidance motivation and, ultimately, avoidance behavior. Exit, leaving the threatened job, occupation, or organization, is the most radical avoidance behavior available to employees (Rusbult et al., 1988), and identity research likewise shows that threatened individuals develop intentions to exit the threatened identity (Petriglieri, 2011; George et al., 2023). Building on coping appraisal, we further propose that technological self-efficacy conditions responses to these identity threats.

As TTAT specifies the behavioral logic, ITT articulates three experiential registers through which harm is felt (value, meanings, enactment); we operationalize as three mediating mechanisms. Integrating the two, we argue that risk perception as a threat appraisal in the context of AI may lead to identity exit since a devalued work identity is experienced as weakened identification (value); an identity whose meaning is no longer sustainable is experienced as a loss of work meaningfulness (meanings); and an identity whose expression is jeopardized is experienced as job insecurity (enactment). We further draw on secondary appraisal to propose that self-efficacy conditions responses to these identity threats: because self-efficacy represents individuals’ beliefs about their capacity to execute the courses of action required to manage prospective situations (Bandura and Wessels, 1997), individuals with high technological self-efficacy should respond to threatened identity value, meaning, and enactment with protection or restructuring responses, whereas low technological self-efficacy should channel the same threats toward withdrawal behavior.

### Occupational replacement risk perception and professional identity

A threat to identity value is an experience in which “the esteem, status, or worth of an identity is being called into question” (George et al., 2023). Identification, the internalization of a group membership or role as a definition of the self, is the natural experiential carrier of value threat. Professional identity refers to the self-concept individuals derive from their occupation, encompassing expertise, status, and a sense of purpose tied to professional roles and group belonging (Ibarra, 1999; Pratt et al., 2006). Within organizational contexts, employees construct and consolidate their professional identities through the performance of role-related tasks (Anteby, 2008), which fulfill job requirements and express their occupational self their occupational self (Pratt et al., 2006). When individuals perceive that their professional expertise and distinctive roles are devalued or overlooked, they are likely to experience identity-related threat (Jussupow et al., 2018). This in line with identity threat theory, which posits that individuals experience threat when valued competencies become less central or recognized (Petriglieri, 2011). Specifically, expertise is a core component of professional identity, and its perceived erosion weakens individuals’ sense of competence and belonging (Ibarra, 1999). As AI increasingly replace tasks requiring specialized knowledge, certain occupations may become less unique or even obsolete, directly threating professional identity (Goto, 2021; Shonhe and Min, 2025). Given that individuals’ perception of their occupation fundamentally shapes how they construct and maintain their professional identity (Sun et al., 2025), perceived risk of occupational replacement may diminish employees’ identification with their expertise and professional status.

Professional identity becomes threatened when people perceive their professional roles to be at risk of marginalization, devaluation, or obsolescence (Steele et al., 2002). As new technologies increasingly takes over tasks requiring specialized knowledge, certain occupations may become less unique or even obsolete (Granulo et al., 2019). This perceived potential of AI to replace or significantly transform human roles elicits competence, value, and existential threats (Frey and Osborne, 2017; Huang and Rust, 2021), collectively undermining employees’ sense of who they are as professionals and creating misalignment between their internal self-concept and external work role enactment. These threats suggest that perceived occupational replacement risk can create a threat that challenges two key dimensions of identification: the sense of personal distinctiveness and the value of group membership (Ashforth et al., 2008; Miscenko and Day, 2016). More specifically, when individuals perceive their occupation as at risk of replacement, they are likely to view their occupation-related expertise as under threat, thereby intensifying negative evaluations of their professional identity.

*Hypothesis 1:* Occupational replacement risk perception is negatively correlated with professional identity.

### Occupational replacement risk perception and work meaningfulness

Meanings reflect how individuals perceive and make sense of an identity (Shamir, 1991). Work meaningfulness is the corresponding psychological state, the perceived significance and value of work arising from one’s job role and organizational membership (Fletcher and Schofield, 2021; Pratt and Ashforth, 2003). An identity meaning threat arises when “the association between an identity and its meanings is unsustainable” (George et al., 2023), that is, when one’s understanding of what the identity means no longer holds. Because identity is a primary source from which work derives meaning (Rosso et al., 2010; Pratt and Ashforth, 2003), work meaningfulness is where such threats are experientially registered. According to identity threat theory, individuals strive to maintain identities that are valued, competent, and continuous (Petriglieri, 2011). However, increasing technological intrusions foster negative perceptions of work roles, eroding uniqueness and significance (Suh and Lee, 2017). This undermines the congruence between occupation and identity, diminishing the sense of meaning derived from work (Walsh and Gordon, 2008). Specifically, employees who perceive AI replacement risk may experience a threat to the importance and uniqueness of their work, thereby undermining their sense of work meaningfulness. When “what it means to be a member of this occupation” is called into question, the work itself loses significance, because work’s significance is substantially constituted by the identity it expresses (Rosso et al., 2010; Wrzesniewski et al., 2003). Hence, erosion of work meaningfulness is the context-appropriate manifestation of an identity meaning threat under AI-driven occupational replacement risk perception.

Work meaningfulness drives not only from the significance and value of one’s work role (Pratt and Ashforth, 2003), but also from workplace opportunities for learning and personal development (Bailey et al., 2019; Fletcher, 2019). AI threatens meaningful work and creates “achievement gaps” by reducing opportunities for achievement in a workplace that gives meaning to the individual (Danaher and Nyholm, 2021). Specifically, occupations satisfy individuals’ basic survival needs as well as higher-order aspirations for self-growth, rendering work central to people’s meaning pursuit and a fundamental source of personal identity (Rosso et al., 2010; Steger et al., 2012). Nevertheless, the increasing displacement of human labor by AI threatens opportunities for achievement in the workplace and undermines the meaning of the work they take on (Scripter, 2025). This undermines the psychological foundations of motivation and engagement, as work no longer satisfies the basic needs of self-enhancement, uniqueness, and authentic self-expression that render work meaningful (Yeoman, 2014). Therefore, higher perceived occupational replacement risk exacerbates threats to work meaningfulness.

*Hypothesis 2:* Occupational replacement risk perception is negatively correlated with work meaningfulness.

### Occupational replacement risk perception and job insecurity

Job insecurity is characterized as pervasive concern over continued employment, accompanied by uncertainty about job stability and continuity (Sverke et al., 2002; Shoss, 2017). It reflects the anticipation of an involuntary deterioration or discontinuation of the employment relationship rather than actual job loss (De Witte, 1999; Sverke et al., 2002). Identity threat theory (Petriglieri, 2011) provides a useful lens for understanding why the perceived risk of occupational replacement gives rise to such insecurity. Identity threat theory holds that individuals construct, express, and sustain valued identities through their work activities and interactions (DeRue and Ashford, 2010), and defines identity threat as an experience appraised as indicating “potential harm to the value, meanings, or enactment of an identity” (Petriglieri, 2011, p. 644). Of relevance here is the enactment form of identity threat, which arises when situational changes limit or prevent an individual from engaging in the activities and interactions through which an identity is expressed (Petriglieri, 2011).

Perceived risk of occupational replacement by AI constitutes precisely such an enactment-form identity threat. When employees anticipate that AI will assume their occupational role, they foresee being deprived of the very tasks, activities, and interactions through which their occupational and organizational identities are expressed and sustained. This anticipation maps closely onto validated measures of enactment threat, which are themselves future-oriented (e.g., “I may no longer be able to engage in activities that express my identity”; George et al., 2023), mirroring the anticipatory nature of job insecurity. Moreover, anticipated replacement renders uncertain employees’ continued membership in the valued social groups through which work identity is lived out, the organization, the occupation, and the employed, thereby constituting an acceptance-based social identity threat (Branscombe et al., 1999). Employees thus fear not only losing their job but also losing the means of expressing who they are at work, and this double jeopardy intensifies their sense of job insecurity. Consistent with this reasoning, technology replacement awareness and robot exposure have been shown to elevate job insecurity (Brougham and Haar, 2020; Lingmont and Alexiou, 2020; Yam et al., 2023). Consequently, the perceived risk of occupational replacement may deepen employees’ sense of job insecurity. Taken together, we hypothesize:

*Hypothesis 3:* Occupational replacement risk perception is positively correlated with job insecurity.

### The mediating mechanism of occupational replacement risk perception and withdrawal behavior

Identity threat theory comprises two main stages: threat generation and response (Petriglieri, 2011). Withdrawal behavior refers to actions employees engage in while still maintaining their organizational membership, typically characterized by reduced effort, disengagement, or diminished dedication in response to perceived threats or stressor (Hanisch and Hulin, 1991). Within Petriglieri’s (2011) identity threat response model, one core strategy is identity protection, which involves distancing oneself from threatening situations as a psychological defense mechanism that preserves self-worth. For example, an employee may withdraw from a collaborative project involving artificial intelligence.

The TTAT suggests that when individuals perceive a threat as personally relevant and severe, they develop a threat-avoidance motivation that predicts avoidance behavior (Liang and Xue, 2009). Such behaviors function as a self-protective mechanism, enabling employees to create distance from environments they deem threatening (Rurkkhum, 2018). Applied to identity processes, the perception that technological change threatens employees’ work identity, by devaluing their expertise, diminishing their distinctiveness, or hindering role enactment, trigger identity threat and subsequent response behaviors. In this sense, withdrawal behavior constitutes a plausible avoidance response, as it serves as a psychological and behavioral means of evading and reducing perceived threats. This study therefore posits that high levels of perceived occupational replacement risk are likely to prompt employees to engage in withdrawal behavior.

Specifically, when employees perceive a risk that their occupations will be replaced by AI, they may interpret it as a threat to the significance and uniqueness of their work, thereby undermining their professional identity and sense of work meaningfulness. A weakened professional identity can foster resistance to new technologies and give rise to negative work behaviors (Mishra et al., 2012). That is, employees who perceive a high level of occupational replacement risk may view their professional status and roles as being threatened, which can lead to withdrawal behaviors aimed at protecting their identity. Meanwhile, work meaningfulness serves as the primary psychological prerequisite for individuals to engage fully in their occupations (Christian et al., 2011). Perceived occupational replacement risk may erode employees’ sense of accomplishment and self-actualization, accelerating the loss of meaning at work and substantially increasing the likelihood of withdrawal behavior. Additionally, job insecurity is a well-established predictor of turnover intention and a central antecedent of work withdrawal behavior (Shoss, 2017). Rather than being merely a self-protective reaction, such insecurity constitutes a key explanatory mechanism through which risk perceptions translate into withdrawal behavior (De Witte, 2005; Jiang and Lavaysse, 2018; Sverke et al., 2002). Taken together, employees who perceive greater occupational replacement risk are more likely to experience a diminished sense of professional identity, an erosion of work meaningfulness, and heightened job insecurity, each of which amplifies the likelihood of withdrawal behavior.

*Hypothesis 4:* Professional identity (a), work meaningfulness (b), and job insecurity (c) will mediate the relationship between occupational replacement risk perception and withdrawal behavior.

### The moderating role of technological self-efficacy

When individuals encounter an identity threat, they evaluate the situation and engage in coping strategies to restore equilibrium (Petriglieri, 2011). Self-efficacy reflects a person’s belief in their capability to perform the actions for achieving intended results (Bandura et al., 1988). TTAT suggested that coping ability affects motivational behavior through perceived self-efficacy (Johnston and Warkentin, 2010; Liang and Xue, 2009). During coping appraisal, individuals evaluate whether they can deal effectively with a threat by considering their own ability to perform those actions. For instance, those who believe they can successfully manage technology tend to accept and use it without fear, whereas those who lack confidence in their coping ability are more likely to avoid it (Celik and Yesilyurt, 2013; Venkatesh and Davis, 1996). In particular, technological self-efficacy is a domain-specific form of self-efficacy based on Bandura’s (1982) larger idea, describing a person’s confidence in navigating, learning, and adapting to digital tools and systems (McDonald and Siegall, 1992). It involves the belief in one’s ability to effectively use technology to accomplish specific tasks or goals (McDonald and Siegall, 1992). It means that if individuals possess sufficient resources to manage perceived threat, they are less likely to view them as a threat to their jobs (Ashford et al., 1989). Thus, when individuals have low technological self-efficacy and perceive the risk of occupation being replaced, they may experience threats to their valued professional identity and work meaning, along with increased unemployment anxiety, as they doubt their ability to cope with the risk.

A strong sense of technology-related self-efficacy may help reduce perceived threats associated with the technology (Alahmad and Robert, 2020; Craig et al., 2019). Employees who feel capable of using AI to perform specific job tasks are less likely to view it as a threat, as their confidence in interacting with the technology fosters acceptance rather than fear (Grundner and Neuhofer, 2021; Złotowski et al., 2017). When individuals have a high level of technological self-efficacy, they believe they can easily learn and use new technologies. This belief can help them integrate AI into their work with a positive mindset, seeing it as a tool to enhance their skills rather than a threat to replace their occupations. This not only enhances professional identity and sustains work meaningfulness but also helps mitigate feelings of job insecurity. In contrast, individuals with low self-efficacy have self-doubts about their abilities, and are likely to exert less effort and cease attempts to resolve threatening problems (Park and Jung, 2015). Thus, employees with lower technological self-efficacy are more likely to perceive occupational replacement risk as threatening to their professional identity and work meaningfulness, inducing feelings of job insecurity and thus increasing their propensity for withdrawal behavior.

*Hypothesis 5:* Technological self-efficacy will moderate the relationship between occupational replacement risk perception and professional identity (a), work meaningfulness (b), and job insecurity (c), such that the relationship will be weaker for those higher in technological self-efficacy.

*Hypothesis 6:* Technological self-efficacy will moderate the indirect effect of occupational replacement risk perception on withdrawal behavior via professional identity (a), work meaningfulness (b), and job insecurity (c), such that the indirect effect is weaker for those higher in technological self-efficacy.

## Overview of studies

To test our hypotheses, we conducted two studies. In Study 1, to address the lack of readily available instruments for assessing perceived risk of occupational replacement, this study adapted items from the existing scales. This adaptation was achieved by following a rigorous scale development procedure to ensure the psychometric validation of the modified instrument. In Study 2, according to a three-wave lagged design, the study analyzes how occupational replacement risk perception influences withdrawal behavior through professional identity, work meaningfulness and job insecurity, as well as the moderating role of technological self-efficacy.

## Study 1

### Method

#### Procedure

##### Item generation

To address the lack of readily available measures of perceived occupational replacement risk, we followed established scale-development procedures (Boateng et al., 2018). Consistent with our conceptual definition grounded in risk perception theory, the construct comprises two dimensions: (a) the perceived likelihood that AI will replace core tasks of one’s occupation, and (b) the perceived severity of the consequences should such replacement occur. Drawing on established measures of risk perception (Wilson et al., 2019) and relevant scales in AI-related settings, such as measures of AI awareness (Brougham and Haar, 2020) and the AI risk perception scale (Wilczek et al., 2025; Wissing and Reinhard, 2018), we selected and adapted conceptually relevant items rather than developing items *de novo*, a procedure that scale-development recommendations recognize as a legitimate deductive approach to item generation (Farh et al., 2006; Hinkin, 1995). To ensure contextual appropriateness, we also drew on informal discussions with employees in AI-exposed occupations to refine item wording and confirm that the adapted items reflected realistic workplace concerns; these conversations informed phrasing only and did not serve as a formal inductive item-generation source. This process yielded an initial pool of eight items.

##### Item refinement

These items were then evaluated by a panel of eight subject matter experts, comprising three assistant professors, three doctoral students, and two corporate human resource professionals. Experts rated each item’s relevance and clarity to the intended dimension on a five-point scale (1 = *not at all*, 5 = *completely*). Mean relevance ratings were 4.15 for perceived likelihood and 4.03 for perceived severity. Within-group agreement (*r_wg_*) was 0.86 for perceived likelihood and 0.82 for perceived severity, exceeding the conventional 0.70 criterion (James et al., 1984), indicating adequate content validity. All items were retained at this stage, with minor wording revisions based on expert feedback. After data collection in the pilot sample, we examined corrected item-total correlations (CITC), applying the conventional retention criterion of 0.40 (Holmbeck and Devine, 2009). Two reverse-worded items fell below this criterion and reduced internal consistency at both the subscale and full-scale levels. This is consistent with methodological evidence that reverse-worded items often induce interpretation errors, response bias, and systematic measurement error (Sonderen et al., 2013). We therefore removed both items, resulting in a final scale of six items in Table 1, which formed the basis for the subsequent reliability and validity analyses.

**Table 1:** Constructs and measurement items of AI-induced occupational replacement risk perception (Study 1).

Items	Factor loading		AVE	CR
Likelihood of perceived occupational replacement risk occurrence			0.78	0.91
The tasks I perform in my occupation could be replaced by AI.	0.91			
My occupation has a high risk of being replaced by AI.	0.90			
Compared to other occupations, my occupation is more susceptible to being replaced by AI in the future.	0.87			
Severity of perceived occupational replacement risk consequence			0.65	0.84
If AI replaced my occupation, the impact on me would be negative.		0.85		
The consequences of AI replacing my occupation would be severe.		0.83		
The replacement of occupations by AI will affect my future.		0.77		
Cumulative variance contribution	34.49%	80.55%		
Cronbach’s *a* coefficient	0.91	0.83		

*N* = 387. CR, composite reliability; AVE, average variance extracted. Responses to items on perceived occupational replacement risk were coded from 1 = very unlikely to 5 = very likely.

### Results

The factor analysis KMO value was 0.80, and Bartlett’s test of sphericity yielded a value of 1470.18 (*p* < 0.001), indicating that the questionnaire has high construct validity and is suitable for further factor analysis.

#### Exploratory factor analysis

Through exploratory factor analysis, two factors were extracted from risk perception: the likelihood of risk occurrence and the severity of the outcome. Table 1 showed that these two-factorial structure explained 80.55% of the variance. The internal consistency test results indicated that the Cronbach’s alpha coefficients for the likelihood risk perception and the consequences risk perception were 0.91and 0.83, both greater than 0.8, indicating that the internal consistency of the scale meets the requirements. Thus, it was determined that the scale consisted of six items and two sub-dimensions.

#### Confirmatory factor analysis

To assess whether the two-dimensional structure derived from exploratory factor analysis was supported by the data, this study conducted confirmatory factor analysis. The results showed that the two-factor model fit the data well (*χ*^2^ = 18.07, *df* = 8, GFI = 0.98, TLI = 0.98, RFI = 0.97, RMSEA = 0.05) and performed better than the one-factor model (*χ*^2^ = 357.18, *df* = 9, GFI = 0.75, CFI = 0.76, TLI = 0.60, RMSEA = 0.32). All fit indices met established thresholds (Hu and Bentler, 1999), supporting the structural validity of the proposed two-dimensional reflective measurement model.

#### Convergent validity and discriminant validity

Table 1 shows the factor loadings, average variance (AVE), and composite reliability (CR). The AVE values for the two factors are 0.78 and 0.65, both of which are above 0.50, thereby providing support for convergent validity (Fornell and Larcker, 1981). CR is used to evaluate the reliability of the structure, with values of 0.91 and 0.84 for the two factors. The reliability coefficients for both structures are greater than 0.70 (Hair et al., 2017), indicating an appropriate level of reliability. Based on the CFA results in Table 1, this study indicates that the scale has good construct validity.

## Study 2

### Method

#### Participants and procedure

To mitigate the potential impact of common method biases, this study conducted a three-wave delay survey to test our hypothesis. Participants were recruited through social media platforms like WeChat, and were encouraged to disseminate the project invitation and survey link to friends or colleagues. Participation was voluntary and anonymous, and respondents were informed of the study’s purpose prior to participation. Participants were required to have full-time employment and at least 1 year of full-time work experience because individuals who did not meet these criteria might lack sufficient experience. To mitigate common method bias and enhance causal inference, we used a three-wave study design, with a four-week interval between each phase. At time 1, perceived occupational replacement risk, technological self-efficacy, and control variables were measured, and 520 surveys were returned. A Time 2, professional identity, work meaningfulness, and job insecurity were collected, and 482 surveys were returned, with a response rate of 92.69%. At Time 3, withdrawal behavior was measured, and 451 surveys were returned, with a response rate of 93.56%. After matching surveys and addressing missing values and outliers, excluding some missing or incomplete answers, we obtained valid data for 443 employees.

Among the 443 employees, 38.8% are male. 7.9% under bachelor degree, 66.8% of employees hold a bachelor’s degree, 25.3% hold a master’s degree and above. Likewise, 37.9% of employees between 20 and 30 years old, 52.4% of employees between 30 and 40 years old, 6.6% between 40 and 50 years old, and 3.2% above 50 years old. Additionally, 29.5% tenure under 5 years, 42.9% tenure between 5 and 10 years, 16.0% tenure between 10 and 15 years, 6.3% tenure between 15 and 20 years, 5.2% tenure above 20 years. The sample spanned six broad occupational categories: professional and technical occupations (*n* = 126, 28.4%), agricultural production and auxiliary occupations (*n* = 100, 22.6%), managerial and organizational-responsibility occupations (*n* = 88, 19.9%), administrative and clerical support occupations (*n* = 67, 15.1%), social production and life service occupations (*n* = 42, 9.5%), and manufacturing and related occupations (*n* = 20, 4.5%). Occupation was included as a control variable in all subsequent analyses. Welch’s one-way analysis of variance indicated that occupational replacement risk perception differed significantly across occupational groups [*F*(5, 123.84) = 4.480, *p* < 0.001].

#### Measures

##### Occupational replacement risk perception

Six items developed in Study 1 were used to measure the occupational replacement risk perception. Participants evaluated the likelihood of their occupation being replaced on a scale of 1 (very unlikely) to 5 (very likely), as well as the impact on them if their occupation is replaced. The items were derived from the scales developed in study 1. In the two-factor structure, the first factor showed a reliability of 0.84, whereas the second factor showed a reliability of 0.83. Cronbach’s α for the overall scale was 0.86.

##### Professional identity

Three items were adapted from the scales developed by Dobrow and Higgins (2005). The scale was assessed using five statements ranked on a 5-point scale anchored at strongly disagree (1) and strongly agree (5). A sample item is “I know who I am, professionally and in my career.” Cronbach’s α for this scale was 0.74.

##### Work meaningfulness

Work meaningfulness was measured with four items adapted from May et al. (2004)’s survey. The scale was assessed using five statements ranked on a 5-point scale anchored at strongly disagree (1) and strongly agree (5). A sample item was “The work I do is meaningful to me.” Cronbach’s α for this scale was 0.75.

##### Job insecurity

Job insecurity was measured using a four-item scale proposed by De Witte (2000). All measures were rated using a Likert response format, ranging from 1 (strongly disagree) to 5 (strongly agree). A sample item is “It is very likely that I will soon lose my job”. This measure showed good internal consistency in the current study (*α* = 0.87).

##### Technological self-efficacy

Five items from McDonald and Siegall (1992) were used to measured Technological self-efficacy. All measures were rated using a Likert response format, ranging from 1 (strongly disagree) to 5 (strongly agree). A sample item is “when I have to learn a new task that is high technology, my first reaction is that I’m sure I can do it”. Cronbach’s α for this scale was 0.83.

##### Withdrawal behavior

Withdrawal behavior was measured using a four-item scale developed by Lehman and Simpson (1992). All measures were rated using a Likert response format, ranging from 1 (strongly disagree) to 5 (strongly agree). The items include “I put less effort into my job than I could have” and “Thoughts of leaving current job”. Cronbach’s α for this scale was 0.82.

##### Control variables

The current study controlled for gender, age, education, and organizational tenure that might influence their professional identity, work meaningfulness and job insecurity (Cheng and Chan, 2008; Van De Mieroop and Clifton, 2012). Considering that objective exposure varies substantially across occupations (Felten et al., 2021), occupation was also included as covariates. This study thus included these control variables in the analysis.

### Results

#### Validity of the measurement model

We assessed the psychometric properties of all measures in the independent Study 2 sample. As shown in Table 2, all standardized factor loadings were statistically significant, and every item loaded on its intended construct. Composite reliability (CR) values ranged from 0.75 to 0.87, all exceeding the recommended 0.70 threshold, and average variance extracted (AVE) values ranged from 0.50 to 0.63, all exceeding the 0.50 criterion (Fornell and Larcker, 1981). These results, obtained in a sample independent of the scale-development sample, provided strong evidence of convergent validity and alleviated concerns regarding the brevity of the initial item pool. Additionally, to assess potential multicollinearity among the predictors, we examined variance inflation factors (VIFs) and tolerance statistics for all variables entered in the regression models. VIF values ranged from 1.02 to 4.50, all below the commonly recommended threshold of 5. These results indicated that multicollinearity was not a concern in the present analyses.

**Table 2:** Item loadings, CRs, and AVEs of measurement model (Study 2).

Variables	Items	Loadings	CR	AVE
Occupational replacement risk perception	ORRP1	0.80	0.85	0.55
	ORRP2	0.76		
	ORRP3	0.71		
	ORRP4	0.70		
	ORRP5	0.70		
	ORRP6	0.72		
Professional identity	PI1	0.70	0.76	0.51
	PI2	0.75		
	PI3	0.72		
Work meaningfulness	WM1	0.75	0.76	0.51
	WM2	0.67		
	WM3	0.74		
	WM4	0.68		
Job insecurity	JI1	0.69	0.87	0.63
	JI2	0.80		
	JI3	0.83		
	JI4	0.84		
Withdrawal behavior	WB1	0.80	0.82	0.53
	WB2	0.73		
	WB3	0.68		
	WB4	0.70		
Technological self-efficacy	TS1	0.74	0.81	0.50
	TS2	0.66		
	TS3	0.68		
	TS4	0.72		
	TS5	0.74		

Given that the scale of occupational replacement risk perception was newly developed in Study 1, we first cross-validated its factor structure in the present independent sample, as recommended in the scale development literature (Worthington and Whittaker, 2006). Confirmatory factor analysis showed that the first-order correlated two-factor model fitted the data well (*χ*^2^ = 140.21, *df* = 8, CFI = 0.93, RMSEA = 0.05) and significantly better than a single-factor model in which all items loaded on a single factor (χ^2^ = 393.71, *df* = 9, CFI = 0.71, RMSEA = 0.31). The two-factor model was significantly better than the single-factor model. These results confirmed the two-dimensional structure of the measure in an independent sample. Furthermore, since the main purpose of Study 2 was to test the hypothesized theoretical model, and our hypothesis was proposed at the overall perceived risk level rather than its dimensional level. As shown in Table 3, the six measured items of occupational replacement risk perception are averaged as the total score of the model testing analysis. At the measurement level, the assumed six factor model fits the data well (*χ*^2^ = 725.07, CFI = 0.93, TLI = 0.92, RMSEA = 0.05), significantly better than the five-factor model that combines perceived occupational replacement risk and job insecurity into one factor (*χ*^2^ = 991.43, CFI = 0.86, TLI = 0.84, RMSEA = 0.07). These results not only indicated conceptual differences among the six variables, but also suggest that that the measurement model exhibited good construct validity.

**Table 3:** Confirmatory factor analysis results (Study 2).

Model	*χ* ^2^	χ^2^/*df*	CFI	TLI	RMSEA
Six-factor model: ORRP, PI, WM, JI, TS, WB	725.07	2.54	0.93	0.92	0.05
Five-factor model: ORRP + JI, PI, WM, TS, WB	991.43	3.43	0.86	0.84	0.07
Four-factor model: ORRP + JI, PI + WM, TS, WB	1188.51	4.05	0.82	0.80	0.08
Three-factor model: ORRP + JI, PI + WM, TS + WB	1451.11	4.90	0.77	0.75	0.09
Two-factor model: PI + WM, ORRP + JI, WB + TS	2297.98	7.71	0.60	0.57	0.12
One-factor model:	2558.11	8.55	0.55	0.51	0.13

*N* =443; ORRP, occupational replacement risk perception; PI, professional identity; WM, work meaningfulness; JI, job insecurity; TS, technological self-efficacy; WB, withdrawal behavior.

#### Hypothesis testing

The descriptive statistics and correlations of the study variables were reported in Table 4.

**Table 4:** Means, standard deviations and correlations among study variables (Study 2).

Variables	Mean	SD	1	2	3	4	5	6	7	8	9	10
1. Gender	1.61	0.49	—									
2. Age	2.96	1.32	−0.04									
3. Education	5.16	0.64	0.04	0.01								
4. OT	3.00	1.27	−0.04	0.87^**^	−0.11^*^							
5. Occupation	3.09	1.78	0.03	0.18^**^	−0.03	0.15^**^						
6. ORRP	3.11	0.92	−0.12^*^	−0.04	−0.24^**^	−0.01	0.04					
7. PI	4.20	0.58	0.11^*^	0.08	0.13^*^	0.07	−0.03	−0.19^**^				
8. WM	4.29	0.53	0.05	0.11^*^	0.10^*^	0.09	−0.05	−0.24^**^	0.59^**^			
9. JI	2.06	0.80	−0.02	−0.30	−0.18^**^	−0.04	0.04	0.64^**^	−0.33^**^	−0.36^**^		
10. TS	4.28	0.49	0.17^**^	0.10^*^	0.12^**^	0.06	0.02	−0.18^**^	0.24^**^	0.39^**^	−0.22^**^	
11. WB	2.00	0.74	−0.03	−0.08	−0.11^*^	−0.07	0.06	0.39^**^	−0.52^**^	−0.65^**^	0.50^**^	−0.30^**^

*N* = 443. ^*^p < 0.05, ^**^p < 0.01. M = Mean, S.D. = Standard Deviation. Gender is coded as 1 = Male and 2 = Female. Age (in years) is coded as 1 = <25, 2 = 26–30, 3 = 31–35, 4=36-40, 5 = 41–45, 6=46-50, 7=>50. Education is coded as 1 = Junior high school or below, 2 = High school, 3=vocational school, 4 = Junior college, 5 = bachelor’s degree, 6 = Master’s degree, 7 = Doctoral degree. Occupational types were coded according to the eight major categories of the Chinese national standard Occupational Classification and Codes (GB/T 6565—2015): 1 = heads of Party and government agencies, people's organizations, social organizations, enterprises, and public institutions; 2 = professional and technical personnel; 3 = clerical and related personnel; 4 = personnel in social production and personal services; 5 = agricultural, forestry, animal husbandry, and fishery production and auxiliary personnel; 6 = manufacturing and related personnel.

As shown in Table 5, occupational replacement risk perception was negatively related to professional identity (*b* = −0.26, SE = 0.05, *p* < 0.001) and work meaningfulness (*b* = −0.22, SE = 0.05, *p* < 0.01), and was positively related to job insecurity (*b* = 0.64, SE = 0.04, *p* < 0.01). These results provide support for Hypotheses 1, 2, and 3. The findings showed that occupational replacement risk perception was positively related to withdrawal behavior (*b* = 0.38, SE = 0.05, *p* < 0.01). Meanwhile, professional identity (*b* = −0.28, SE = 0.07, *p* < 0.01) and work meaningfulness (*b* = −0.35, SE = 0.06, *p* < 0.01) were negatively related to withdrawal behavior, whereas job insecurity (*b* = 0.21, SE = 0.08, *p* < 0.01) was positively associated with withdrawal behavior. In Table 4, the indirect effects of occupational replacement risk perception on withdrawal behavior through professional identity (*b* = 0.08 [95% CI = 0.04, 0.12]), work meaningfulness (*b* = 0.09 [95% CI = 0.04, 0.13]), and job insecurity (*b* = 0.14 [95% CI = 0.08, 0.19]) were statistically significant. These results provide support for Hypotheses 4.

**Table 5:** Regression analysis result (Study 2).

Variable	Professional identity		Work meaningfulness		Job insecurity		Withdrawal behavior	
	Model 1	Model 2	Model 3	Model 4	Model 5	Model 6	Model 7	Model 8
	*β* SE	*β* SE	*β* SE	*β* SE	*β* SE	*β* SE	*β* SE	*β* SE
Gender	0.10 (0.09)	0.4 (0.09)	0.03 (0.09)	−0.02 (0.09)	0.05 (0.07)	0.07^**^ (0.07)	0.02 (0.09)	0.04 (0.07)
Age	−0.04 (0.07)	−0.05 (0.07)	0.07 (0.07)	−0.04 (0.07)	0.15^*^ (0.06)	0.19^**^ (0.06)	−0.04 (0.07)	−0.03 (0.05)
Education	0.03(0.08)	0.04 (0.07)	0.05 (0.08)	0.01 (0.07)	−0.05 (0.06)	−0.04 (0.06)	−0.02 (0.07)	0.03 (0.05)
Organizational tenure	0.13 (0.08)	0.11 (0.07)	0.03 (0.08)	0.10 (0.07)	−0.17^*^ (0.06)	−0.21^**^ (0.06)	−0.03 (0.07)	0.03 (0.05)
Occupation	−0.04 (0.03)	−0.04 (0.03)	−0.07 (0.03)	−0.07 (0.03)	−0.01 (0.02)	0.01 (0.02)	0.06 (0.03)	0.02 (0.02)
ORRP	−0.17^**^ (0.05)	−0.16^**^ (0.05)	−0.22^**^ (0.05)	−0.20^**^ (0.04)	0.59^**^ (0.04)	0.57^**^ (0.04)	0.38^**^ (0.05)	0.11^**^ (0.04)
TS		0.16^**^ (0.05)		0.30^**^ (0.05)		−0.09^*^ (0.04)		
ORRP × TS		0.15^**^ (0.05)		0.22^**^ (0.05)		−0.12^**^ (0.04)		
PI								−0.15^**^ (0.06)
WM								−0.35^**^ (0.06)
JI								0.21^**^ (0.04)
*R^2^*	0.09	0.21	0.06	0.22	0.42	0.45	0.14	0.56
*△R^2^*		0.12		0.16		0.03		0.42
*F*	8.67^**^	13.79^**^	6.44^**^	18.55^**^	63.89^**^	50.93^**^	15.81^**^	71.42^**^

*N* = 443. ^*^p < 0.05, ^**^p < 0.01. ORRP is occupational replacement risk perception.

As shown in Table 5, the interaction of occupational replacement risk perception and technological self-efficacy was significant and positive in predicting professional identity (*b* = 0.17, SE = 0.05, *p* < 0.01) and work meaningfulness (*b* = 0.22, SE = 0.05, *p* < 0.01). The patterns of these interactions were plotted in Figures 2, 3, providing support for Hypothesis 5. Simple slope tests indicated that the negative association between occupational replacement risk perception and professional identity was strong when technological self-efficacy was low (−1 SD; *b* = −0.59, *p* < 0.01) but became weak and non-significant when technological self-efficacy high (+1 SD; *b* = −0.26, *ns*). Moreover, the negative association between occupational replacement risk perception and work meaningfulness was strong at low technological self-efficacy (−1 SD; *b* = −0.65, *p <* 0.01) but weak and non-significant at high technological self-efficacy (+1 SD; *b* = −0.10, *ns*). These results indicated that technological self-efficacy buffers the negative relationships of occupational replacement risk perception with professional identity and work meaningfulness. In addition, the interaction occupational replacement risk perception and technological self-efficacy was significant and negative in predicting job insecurity (*b* = −0.12, SE = 0.04, *p* < 0.05). The pattern of this interaction was plotted in Figure 4. Simple slope tests revealed that the positive association between occupational replacement risk perception and job insecurity was stronger when technological self-efficacy was low (−1 SD; *b* = 0.90, *p* < 0.01) than when technological self-efficacy was high (+1 SD; *b* = −0.50, *p* < 0.01), again indicating a buffering role of technological self-efficacy.

**Figure 2:**
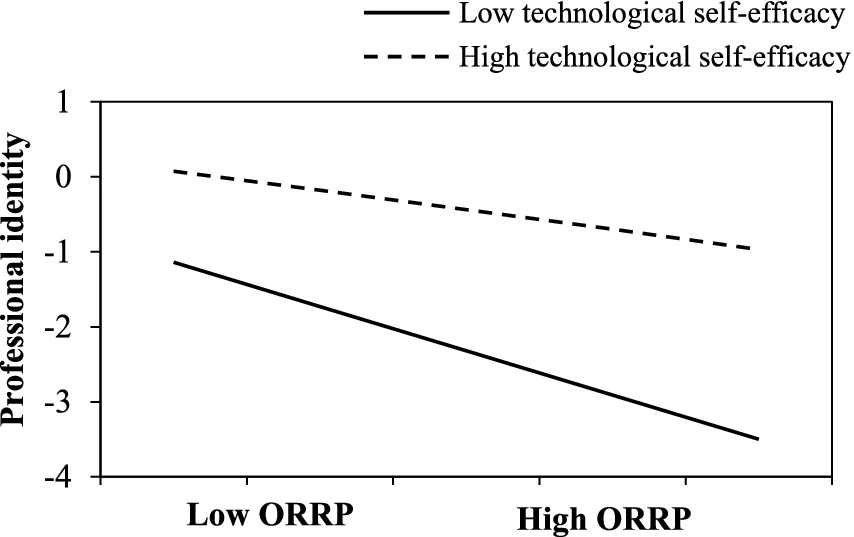
The moderating role of technological self-efficacy on the relationship between ORRP and professional identity.

**Figure 3:**
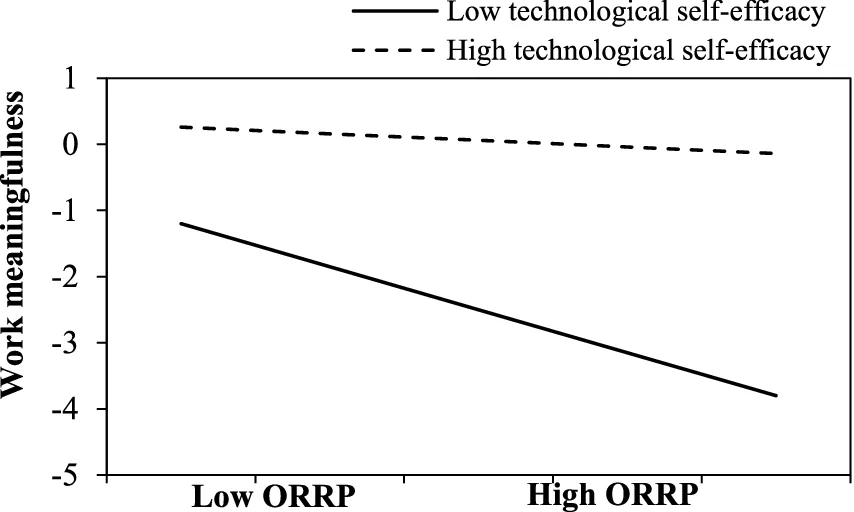
The moderating role of technological self-efficacy on the relationship between ORRP and work meaningfulness.

**Figure 4:**
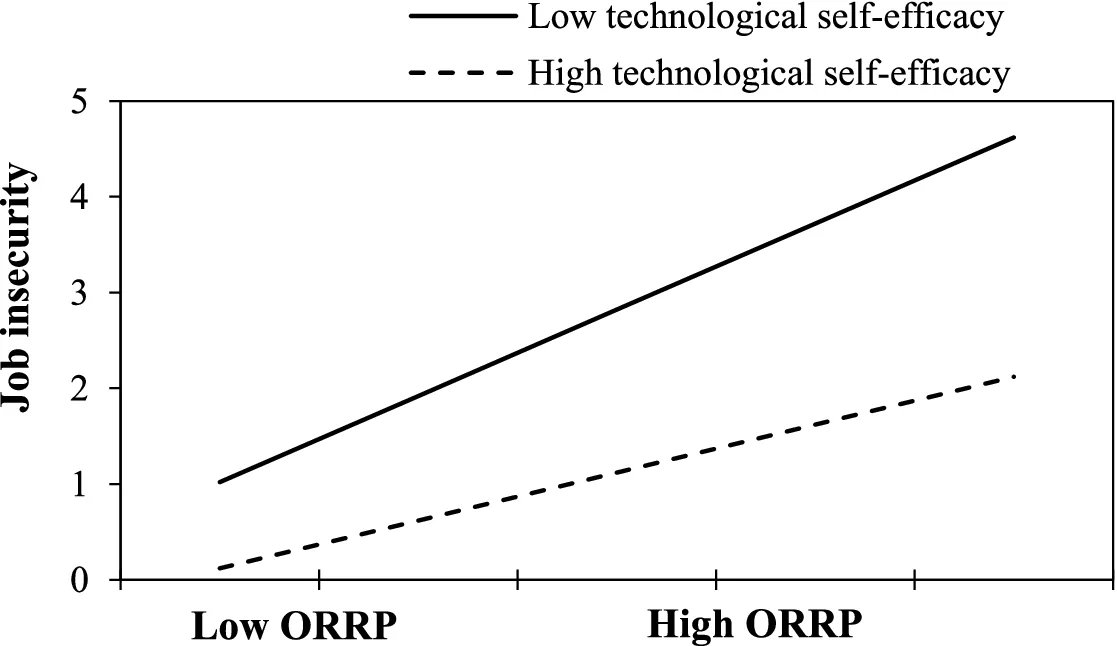
The moderating role of technological self-efficacy on the relationship between ORRP and job insecurity.

**Table 6:** Indirect and conditional indirect effects (Study 2).

Indirect and conditional indirect effects	*B*	*SE*	95%CILI	95%CIUL
Indirect effects				
Indirect effect of professional identity	0.08	0.03	0.04	0.15
Indirect effect of work meaningfulness	0.09	0.02	0.04	0.13
Indirect effect of job insecurity	0.14	0.03	0.01	0.19
*Conditional indirect effects*				
For professional identity				
−1 SD	0.15	0.05	0.06	0.25
+1 SD	0.01	0.03	−0.05	0.08
Difference	−0.07	0.03	−0.13	−0.01
For work meaningfulness				
−1 SD	0.26	0.06	0.14	0.37
+1 SD	−0.02	0.04	−0.09	0.06
Difference	−0.14	0.04	−0.21	−0.06
For job insecurity				
−1 SD	0.33	0.062	0.22	0.46
+1 SD	0.23	0.040	0.15	0.31
Difference	−0.05	0.020	−0.10	−0.02

Bootstrap sample size 10,000. CI confidence interval.

Table 6 indicated that the indirect effects of perceived occupational replacement risk on withdrawal behavior through professional identity were more positive under lower technological self-efficacy (*b* = 0.25, [95%CI = 0.13, 0.37]) compared with higher technological self-efficacy (*b* = 0.04, [95%CI = −0.03, 0.13]). The difference between the indirect effects was also significant (*b* = −0.11, [95%CI = −0.18, −0.02]). We found that the indirect effects of occupational replacement risk perception on withdrawal behavior via work meaningfulness were stronger under lower technological self-efficacy (*b* = 0.26, [95%CI = 0.14, 0.37]) compared to higher technological self-efficacy (*b* = −0.02, [95%CI = −0.09, 0.06]). The difference between the indirect effects were significant (*b* = −0.14, [95% CI = −0.21, −0.06]). In addition, results further indicated that the indirect effects of occupational replacement risk perception on withdrawal behavior via job insecurity were more positive under lower technological self-efficacy (*b* = 0.33, [95% CI = 0.22, 0.46]) compared to higher technological self-efficacy (*b* = 0.23, [95% CI = 0.15, 0.31]). The difference between the indirect effects was significant (*b* = −0.05, [95% CI = −0.10, −0.02]). Thus, Hypothesis 6 was supported.

## Discussion

### Theoretical contributions

First, this paper contributes to the existing literature by addressing an underexplored gap concerning AI-induced occupational replacement risk perception. It not only underscores the significance of individuals’ subjective perception of occupational replacement arising from AI but also responds to scholars’ calls to explore how people respond to such technological replacement. Whereas prior research in technological contexts has mainly treated work change and technological autonomy as key antecedents of identity threat, the present study highlights the risk perceptions related to occupational replacement originating from the external environment as a critical source of identity threat. Moreover, this study contributes to identity threat theory by elaborating a distinctive antecedent of identity threat, extending its applicability to technology-enabled work settings, and empirically validating the dimensional framework of Petriglieri (2011) and George et al. (2023). In addition, while TTAT has been employed to examine individuals’ threat perceptions of emerging technologies that generate potential risks, we extend this theoretical lens by examining behavior specifically through perceived risk of occupational replacement. Therefore, the study provides new insights into the perspective of identity threat in the AI context and expands theoretical understanding by linking AI-induced risk perception of occupational replacement to behavior.

Second, the current study investigated how risk perception triggers behavior by examining the roles of professional identity, work meaningfulness, and job insecurity, thereby contributing to the enrichment of relevant research literature in the field. The results indicate that perceived occupational replacement risk reduces individuals’ professional identity and work meaningfulness, while increasing their job insecurity. Consistent with previous studies suggesting that artificial intelligence may exert adverse impacts on individuals’ work roles, our findings validate and supplement such perspectives (Shonhe and Min, 2025). Meanwhile, this study fills a research gap in empirical literature on occupation and meaning in the context of AI. While recent studies have provided ethical analysis and review of how AI supports or undermines meaning (Callari and Puppione, 2025), such studies have predominantly been conceptual and lack rigorous empirical testing. This paper also provides empirical evidence that the perception of occupational replacement risk reduces the sense of work meaningfulness (Zhang and Amos, 2026), thus addressing the empirical void in this domain. Additionally, our findings are consistent with empirical research in which AI awareness positively predicts insecurity, which subsequently mediates adverse outcomes such as increased turnover intention (Kang et al., 2024). Overall, this study deepens understanding of how occupational replacement risk perception influences professional identity, work meaningfulness, and job insecurity, thereby helping to fill an empirical gap in the existing literature.

Third, our findings suggest that technological self-efficacy represents a critical boundary condition under which the effects of perceived occupational replacement risk on professional identity, work meaningfulness, and job insecurity vary in strength. The results emphasize that for employees with higher technological self-efficacy, the negative impacts of risk perception on professional identity and work meaningfulness, as well as its positive impact on job insecurity, are weakened. Our results not only provide additional support for the role of technological self-efficacy in specific domains, but also highlight its importance as a coping capability that helps employees manage the challenges associated with technological shocks. Furthermore, our findings are consistent with prior research suggesting that technological self-efficacy functions as an important personal resource, such that employees with higher technological self-efficacy are more capable of mastering AI tools and developing proactive behaviors. As a recent study indicated, domain-specific self-efficacy buffers the negative effects of technology by mitigating adverse psychological and behavioral outcomes linked to technostress and perceived role erosion (Song et al., 2025). Thus, the current research supplements the existing research on technological self-efficacy by providing deeper insights into how employees cope with the increasing risk of AI-induced occupational replacement.

### Practical implications

This study also provides inspiration for practice. Occupational psychologists should take the lead in conducting systematic assessments of technology-induced occupational threats, identify high-risk groups (e.g., employees in highly automatable positions), providing a scientific basis for subsequent targeted interventions. For individuals, the perception of technology needs to shift from viewing it as a threat to their occupations to seeing it as a tool that enhances capabilities and generates new opportunities. As our results indicate that technological self-efficacy can serve as a coping ability, individuals need to be fully prepared for the introduction of AI by building confidence and a positive mindset toward emerging technologies, helping to reduce potential threats to their sense of identity. Moreover, research has shown that technological factors pose a significant threat to workplace identity and career extension (Shonhe and Min, 2025), and are related to individual skills and effort. To cope with identity threats, individuals need to continuously upgrade their skills, adopt new technologies, and enhance their technological and professional adaptability.

From an organizational perspective, managers and organizations play a crucial role in responding to threats. On the one hand, managers need to be more committed to understanding the impact of AI on humanity, reduce the negative impacts of AI through diversity and inclusion policies and strategies, and minimize employee behavior caused by perceived occupational replacement risk. On the other hand, when new technological changes are integrated into business practices, organizations should involve employees as much as possible to ensure maximum collaboration. Therefore, organizations should invest in targeted professional development, upskilling, and reskilling programs to enhance employees’ technological self-efficacy. From this perspective, organizations could help employees better cope with the risk of occupational replacement by creating healthy work environments and providing appropriate vocational skills training programs to enable employees to adapt to the evolving, technology-driven workplace.

### Limitations and future directions

Several limitations of this study warrant attention. First, all study variables were assessed via self-report, rendering the data susceptible to common method bias and social desirability effects. Although the three-wave time-lagged design partially mitigated these concerns, it could not eliminate them entirely. Second, although we developed the scale following established scale-development procedures (Boateng et al., 2018; MacKenzie et al., 2011), its incremental validity remains untested. Future research should therefore measure these constructs concurrently to examine whether occupational replacement risk predicts relevant outcomes beyond established awareness constructs such as STARA awareness and AI awareness, thereby providing stronger evidence for the construct’s unique contribution. Third, the data remain observational, and because the mediators and withdrawal behavior were not measured at baseline, we could not control for their baseline levels or rule out reverse causation; future research could measure all focal variables at every wave or adopt cross-lagged panel, experimental, or quasi-experimental designs. Fourth, while occupation was controlled for, we did not test the robustness of the model across occupational types, and our data captured no industry-level information; future research could address these limitations by employing larger, stratified samples to examine occupational- and industry-level heterogeneity.

## Data Availability

The raw data supporting the conclusions of this article will be made available by the authors, without undue reservation.
